# Complex gene expansion of the *CYP2D* gene subfamily

**DOI:** 10.1002/ece3.4568

**Published:** 2018-10-12

**Authors:** Ping Feng, Zhijun Liu

**Affiliations:** ^1^ Key Laboratory of Ecology of Rare and Endangered Species and Environmental Protection (Guangxi Normal University) Ministry of Education Guilin China; ^2^ Guangxi Key Laboratory of Rare and Endangered Animal Ecology Guangxi Normal University Guilin China; ^3^ College of Life Sciences Guangxi Normal University Guilin China

**Keywords:** *CYP2D* subfamily, diet, evolution, gene expansion, P450

## Abstract

Cytochrome P450 (CYP) superfamily genes encode enzymes that play a role in metabolizing endogenous compounds and in detoxifying exogenous chemicals. The CYP2D subfamily is a member of the CYP2 family, and its gene expansion in herbivores is presumably linked with the need to detoxify abundant plant toxins in the diet, which indicates that *CYP2D* gene expansion is associated with dietary preferences. To test this hypothesis, the dietary information and *CYP2D* gene number for 73 vertebrates from different taxonomic groups including 22 mammals, 49 birds, 1 reptile, and 1 amphibian were collected, and correlation analysis and ANOVA were conducted. The results showed that most species (45/73) had only one *CYP2D* gene, despite their different diets, and dietary preferences were not correlated with *CYP2D* gene numbers. Specifically, the majority of birds and 7 mammals had only 1 *CYP2D* gene, and the *CYP2D* gene number of mammals ranged from 1 to 11, irrespective of their feeding habits. Species with a *CYP2D* gene number ≥5 included carnivores, herbivores, and omnivores. Furthermore, statistical analyses revealed that no significant correlation existed between dietary preferences and *CYP2D* gene number, and there was no significant *CYP2D* gene number variation among species with different dietary preferences, regardless of whether all vertebrates or specific lineages were considered. Furthermore, gene dynamics which indicated by gene duplication events and loss events showed that *CYP2D* gene number variation had no relationship with diet, suggesting that diet was not a driving force of *CYP2D* gene expansion and that *CYP2D* gene expansion was more complex than previously recognized.

## 1 INTRODUCTION

Most plant species can produce complex toxic secondary metabolites which have anti‐herbivore effect; these metabolites serve as sexual hormones, metal‐transporting agents, differentiation effectors, and so on (Demain & Fang, 2000; Sullivan, Hagen, & Hammerstein, 2008). In addition, among the plant secondary metabolites, alkaloids, such as cocaine and nicotine, are potent neurotoxins that evolved to prevent herbivores from consuming plants (Karban & Baldwin, 1997; Roberts & Wink, 1998; Sullivan et al., 2008). As countermeasures, herbivores have evolved physiological mechanisms to defend against or minimize the harmful effects of plant secondary chemicals (McArthur, Hagerman, & Robbins, 1991). Examples include evolving chemosensory receptors that prevent the animals from absorbing toxic or harmful substances, forming symbiotic relationships with microbes to extract nutrients from plants, forming cellular membranes for multidrug transport, and so forth (Karban & Agrawal, 2002). Among these strategies, perhaps the most general one is evolving enzymes that can detoxify plant secondary chemicals (Karban & Agrawal, 2002; Sullivan et al., 2008).

Enzymes in the cytochrome P450 (CYP) superfamily can protect animals against the attack of exogenous compounds; thus, these enzymes are important for survival. Cytochrome P450 (*CYP*) genes encode enzymes (Palmer & Reedijk, 1991) catalyzing the oxidation of exogenous and noxious environmental chemicals, such as drugs, steroids, and carcinogenic compounds present in food (Yasukochi & Satta, 2015). They are distributed widely across various organisms (Nelson, 2009), from archaea, bacteria, and viruses to higher plants and animals (Gotoh, 2012).

According to the difference in substrates, CYPs can be categorized into two types: the biosynthesis type (B‐type) and the detoxification type (D‐type) (Gotoh, 2012; Nebert & Dalton, 2006; Rezen, Debeljak, Kordis, & Rozman, 2004). In humans, the D‐type is responsible for the detoxification of xenobiotics such as aromatic compounds, plant alkaloids, and especially drugs; the B‐type is generally involved in endogenous processes, for example, the biosynthesis of physiologically active chemicals such as steroids, hormones, and cholesterols (Kawashima & Satta, 2014; Martignoni, Groothuis, & de Kanter, 2006). Although involved in steroid and eicosanoid metabolism, mammalian CYP1‐4s are usually viewed as detoxification enzymes (Gotoh, 2012; Nebert & Dalton, 2006).

Among vertebrate CYP families, CYP2 family is the largest and most diverse (Nelson, 2003; Nelson et al., 2004). The CYP2 family plays an important role in metabolizing various endogenous and exogenous chemicals (Lee et al., 2008; Wang & Tompkins, 2008). Due to their ability to metabolize a wide range of chemical compounds, including many clinically important drugs such as fluoxetine, the *CYP2D* genes of mammals have fascinated to many researchers and have received a considerable amount of attention (Kirischian, McArthur, Jesuthasan, Krattenmacher, & Wilson, 2011). Although the CYP2 family includes 29 subfamilies, only a few subfamilies are widely distributed across vertebrate taxa, and *CYP2D* subfamily members have mainly been identified in amphibians, birds, and mammals (Kirischian et al., 2011; Nelson, 2009). CYP2D isoform is the first one exhibiting polymorphism, and it functions in the monooxygenation of diverse substrates, such as β‐blockers, antidepressants, dextromethorphan, antiarrhythmics (Hiroi, Chow, Imaoka, & Funae, 2002; Martignoni et al., 2006).

The human *CYP2D* gene subfamily comprises C*YP2D6*, C*YP2D7,* and C*YP2D8*, the latter two of which are often pseudogenes in some species (Nelson, 2009). The CYP2D6 enzyme of human has a high affinity for alkaloids, and it can detoxify them (Fonne‐Pfister & Meyer, 1988). Although the proportion of the CYP2D6 enzyme accounts for only ~4% of the total P450 content of the liver, the enzyme is very important in that its substrates constitute approximately 25% of frequently prescribed drugs (e.g., antiarrhythmics, β‐blockers, and antidepressants) (Ingelman‐Sundberg, 2005; Yasukochi & Satta, 2015; Zuber, Anzenbacherova, & Anzenbacher, 2002).

*CYP2D* gene number refers to the number of gene belonging to the *CYP2D* gene subfamily; for example, if a species has *CYP2D6*,* CYP2D7*, and *CYP2D8* then this species has 3 *CYP2D* genes. The number of *CYP2D* genes varies among species. Although most birds have a single *CYP2D* gene, species of amphibians and primary mammalian orders have many *CYP2D* genes and exhibit an independent expansion of the *CYP2D* subfamily. For example, primates have 2–3 *CYP2D* genes, whereas in rodents, rabbits, and horses, the *CYP2D* gene numbers are 5–7, 5, and 6, respectively (Cooke, Bligh, Cybulski, Ioannides, & Hall, 2012; Uno, Iwasaki, Yamazaki, & Nelson, 2011; Uno, Uehara, Kohara, Murayama, & Yamazaki, 2010; Yasukochi & Satta, 2015). It has been proposed that the expansion of *CYP2D* subfamily genes could be associated with feeding habits and with plant toxins (e.g., alkaloids) metabolism (Fonne‐Pfister & Meyer, 1988; Yasukochi & Satta, 2015). Generally, herbivores encounter more abundant plant toxins than omnivores and carnivores, which have fewer plant species in their diets. Additionally, the *CYP2D* subfamily, especially the *CYP2D6* gene, is responsible for plant toxin detoxification. Thus, the study here intended to explore whether dietary preference was a driving force for *CYP2D* subfamily gene expansion across vertebrates. This problem was divided into several questions: (a) Is the gene number of *CYP2D* subfamily associated with diet? (b) Do the *CYP2D* gene numbers vary according to dietary preference? (c) Is the correlation mentioned in (a) or the relationship between gene number variance and diet mentioned in (b) lineage specific? and (d) Are the dynamics of *CYP2D* gene number associated with diet? To answer these questions, the number of *CYP2D* genes and dietary information were collected across the vertebrates, and corresponding statistical analyses and gene dynamics surveys were performed.

## 2 MATERIALS AND METHODS

### 2.1 Data sources

The data of *CYP2D* gene numbers were obtained from published papers (He, Chen, Yang, & Zhou, 2016; Nelson, 2009; Yasukochi & Satta, 2011) (see the Supporting Information [Link]) and multiple databases, including those of Ensembl (http://www.ensembl.org/), the National Center for Biotechnology Information (http://www.ncbi.nlm.nih.gov/genome/), the Cytochrome P450 Homepage (http://drnelson.uthsc.edu/CytochromeP450.html), and The Human Cytochrome P450 (CYP) Allele Nomenclature Database (http://www.cypalleles.ki.se/). The number of *CYP2D* genes selected was based on the criterion that the number was reported in publicly available literature. First, the database mentioned in Nelson (2009) was consulted due to its comprehensive summary of *CYP2D* genes from different taxonomic groups of species. Then, the newest literature on *CYP2D* genes and their related references were searched to obtain more information about *CYP2D* gene number. When the gene numbers conflicted with each other, the sequence present in the literature was checked, and more literature was examined to decide which one was true. In addition, dietary information was collected from literature and database resources. According to their dietary preferences, animals can be classified into three kinds: carnivores, omnivores, and herbivores, which are often based on the 90% rule (Harestad & Bunnell, 1979). In brief, a species is viewed as herbivorous (or carnivorous) when its diet contains ninety percent or more plant (or animal) tissue, and the others are considered omnivorous. The feeding habit was mainly obtained from the Animal Diversity Web (http://animaldiversity.org, last accessed September 21, 2017), and Li and Zhang (2014), Wang and Zhao (2015), etc. (see Supporting Information). The species tree was reconstructed by referring to Murphy, Pevzner, and O'Brien (2004), Zhao, Li, and Zhang (2015), and Jarvis et al. (2014). Numbers of total genes, intact genes, and pseudogenes of *CYP2D* subfamily members and diet were assigned to the corresponding species on the species tree.

### 2.2 Statistical analysis

To examine whether the number of *CYP2D* genes was related to dietary preference or not, a correlation analysis was performed. In addition, to test whether *CYP2D* gene number varied according to the diet of the species, analysis of variance (ANOVA) was conducted. Both analyses were carried out by using SPSS 16.0 (SPSS Inc., Chicago, IL). To test whether the correlation or gene number discrepancy was lineage specific, correlation analysis and ANOVA were also carried out in a bird group and a mammal group, respectively. However, species that are phylogenetically related are apt to resemble each other in most traits (Blomberg, Garland, & Ives, 2003), which results in non‐independence of data in the statistical analysis. Thus, the phylogenetic comparative method was used to solve this problem by removing the effect of phylogeny (Felsenstein, 1985; Harvey & Pagel, 1991). In this study, the PDAP module of Mesquite software (Maddison & Maddison, 2017) and the phytools package (Revell, 2012) of R 3.4.3 (R Core Team, 2016) were used to remove the effect of phylogeny during the statistical analyses.

### 2.3 Survey of gene duplication and gene loss events

To explore whether the dynamics of *CYP2D* gene number have a relationship with diet, gene duplication events and gene loss events were surveyed. First, amino acid sequences of the *CYP2D* subfamily members mentioned above were downloaded: from these, the pseudogenes were excluded due to their great divergence. In addition, because of the failure to obtain the gorilla CYP2D7 sequence, the survey here did not include the gorilla. Second, a neighbor‐joining tree of the CYP2D sequences was created by MEGA 6.0 (Tamura, Stecher, Peterson, Filipski, & Kumar, 2013) using the default setting. Third, the protein tree was compared to the species tree reconstructed before using Notung 2.9 (Durand, Halldorsson, & Vernot, 2006) to locate the duplication and loss events.

## 3 RESULTS

**Figure 1:**
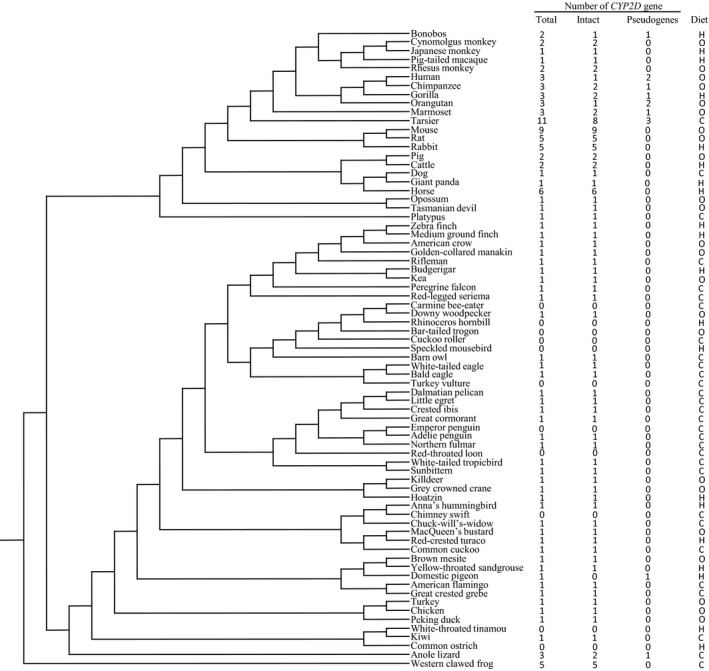
*CYP2D* gene numbers and diet information of 73 vertebrates used in this study. C: carnivorous; H: herbivorous; O: omnivorous

**Figure 2:**
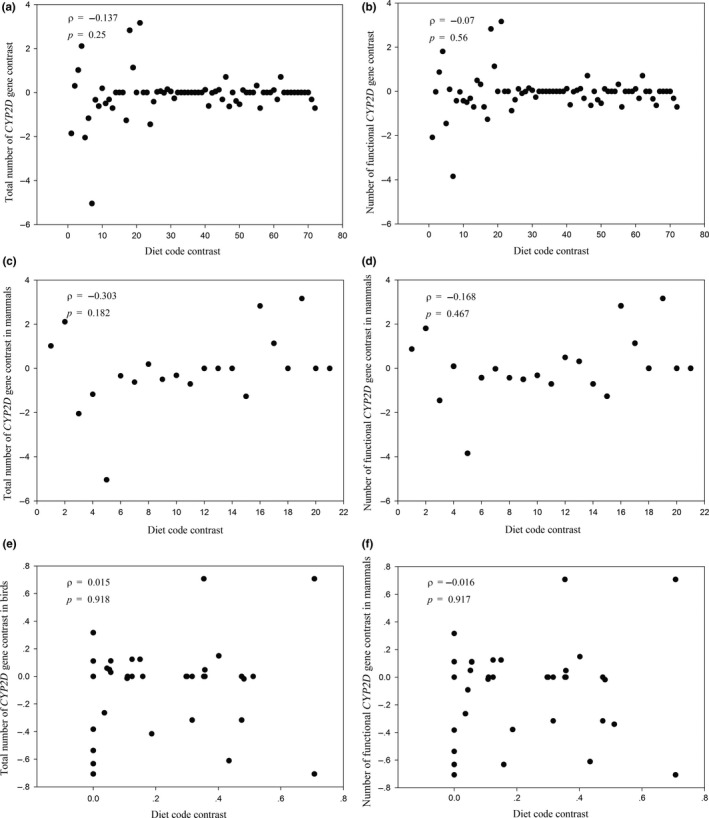
Phylogenetically independent contrasts (PICs) of dietary preferences had no correlation with PICs of *CYP2D* gene number. (a) PICs of total number of *CYP2D* gene didn't correlate with that of diet code in 73 species; (b) PICs of number of functional *CYP2D* gene had no correlation with that of diet code in 73 species; (c) PICs of total number of *CYP2D* gene didn't correlate with that of diet code in 22 mammals; (d) PICs of number of functional *CYP2D* gene had no correlation with that of diet code in 22 mammals; (e) PICs of total number of *CYP2D* gene didn't correlate with that of diet code in 49 birds; (f) PICs of number of functional *CYP2D* gene had no correlation with that of diet code in 49 birds. Each species was coded with 0 (carnivorous), 0.5 (omnivorous), or 1 (herbivorous), according to the potential toxins content in their food. The Spearman's rank correlation coefficient (*ρ*) was used to evaluate the association with a two‐tailed *p*‐value

Seventy‐three species from 46 orders of vertebrates that had both *CYP2D* gene number and dietary information were obtained (Figure 1 and Supporting Information), including 22 mammals from 9 orders, 49 birds from 35 orders, 1 reptile, and 1 amphibian. Most species had at least 1 functional *CYP2D* gene. Specifically, in mammalian species, the *CYP2D* gene number ranged from 1 to 11, and 7 species had only 1 *CYP2D* gene, including carnivorous, herbivorous, and omnivorous species; in avian species, the number of *CYP2D* genes was either 1 or 0, regardless of their feeding preferences; and across the vertebrates, the species for which the *CYP2D* gene number was ≥5 included carnivores, herbivores, and omnivores. The results are shown in Figure 1. The numbers of total *CYP2D* genes, functional genes and pseudogenes ranged from 0 to 11, 0 to 9, and 0 to 3, respectively. Many species had only 1 intact *CYP2D* gene and 0 pseudogenes. In terms of gene expansion, 8, 9, and 6 functional genes existed in the carnivore (tarsier), omnivore (mouse), and herbivore (horse), respectively. Therefore, in a preliminary observation, there was no obvious relationship between *CYP2D* gene number and diet. To confirm this observation, correlation analysis was carried out. In detail, the dietary preference of a species was coded as 1 (herbivorous), 0.5 (omnivorous), or 0 (carnivorous), and correlation analysis between dietary codes and *CYP2D* gene numbers was performed. Because phylogenetic inertia, which means that more closely related species have more similar traits (Feng, Zhao, & Lu, 2015; Fisher & Owens, 2004), can result in data non‐independence, phylogenetically independent contrasts (PIC) (Felsenstein, 1985) were employed to remove this effect by using Mesquite software (Maddison & Maddison, 2017). Specifically, the 73 phylogenetically correlated data points were converted into 72 PICs using a species tree of the 73 species. Since the data did not fit the standard normal distribution (*p *< 0.05, Kolmogorov–Smirnov test), the nonparametric Spearman's rank correlation coefficient (*ρ*) was used to assess the correlation (Wang & Zhao, 2015). The result revealed no correlation between the number of *CYP2D* genes and diet. For the total number of *CYP2D* genes, the correlation coefficient and *p*‐value for the PICs of the dietary code and the *CYP2D* gene numbers were *ρ* = −0.137 and *p *= 0.25, respectively, and for functional genes, they were *ρ* = −0.07 and *p *= 0.56, respectively. When only considering mammals, the correlation coefficient and *p*‐value for the PICs were *ρ* = −0.303 and *p *= 0.182 (total) and *ρ* = −0.168 and *p *= 0.467 (functional), respectively. In addition, in birds, they were *ρ* = 0.015 and *p *= 0.918 (total) and *ρ* = −0.016 and *p *= 0.917 (functional), respectively. The results of correlation analyses are shown in Figure 2.

To further clarify whether dietary preferences drove the expansion of *CYP2D* genes, analysis of variance (ANOVA) of dietary code and *CYP2D* gene numbers was performed. Since the data did not fit the standard normal distribution (*p *< 0.05, Kolmogorov‐Smirnov test), the Jonckheere–Terpstra test implemented in nonparametric tests was used to assess the differences. The result demonstrated that species with different dietary preferences did not significantly vary in the number of *CYP2D* genes for the total number of *CYP2D* genes (*p *= 0.782) or the number of functional *CYP2D* genes (*p *= 0.519). When only considering mammals, the *p*‐values for total *CYP2D* genes and functional *CYP2D* genes were 0.648 and 0.798, respectively. In birds, they were 0.303 and 0.115, respectively. After removing the phylogenetic similarity by using the R package, the conclusion was similar, with *p *= 0.733 for total *CYP2D* genes and *p *= 0.77 for functional *CYP2D* genes in the vertebrate group; in mammals, they were 0.69 and 0.858, respectively, and in birds, they were 0.488 and 0.314, respectively. The results of standard ANOVA and phylANOVA were clearly the same, and neither was significant. This result may have been due to the limited number of species, which is supported by the suggestion of Rohlfs and Nielsen (2015) that although standard ANOVA cannot analyze trait data without considering similarity between closely related species, such similarity has a limited effect on small samples sizes. In sum, species with different dietary preferences did not differ significantly in *CYP2D* gene number, regardless of the lineage level at which the data were analyzed.

### 3.1 Gene duplication and loss events

**Figure 3:**
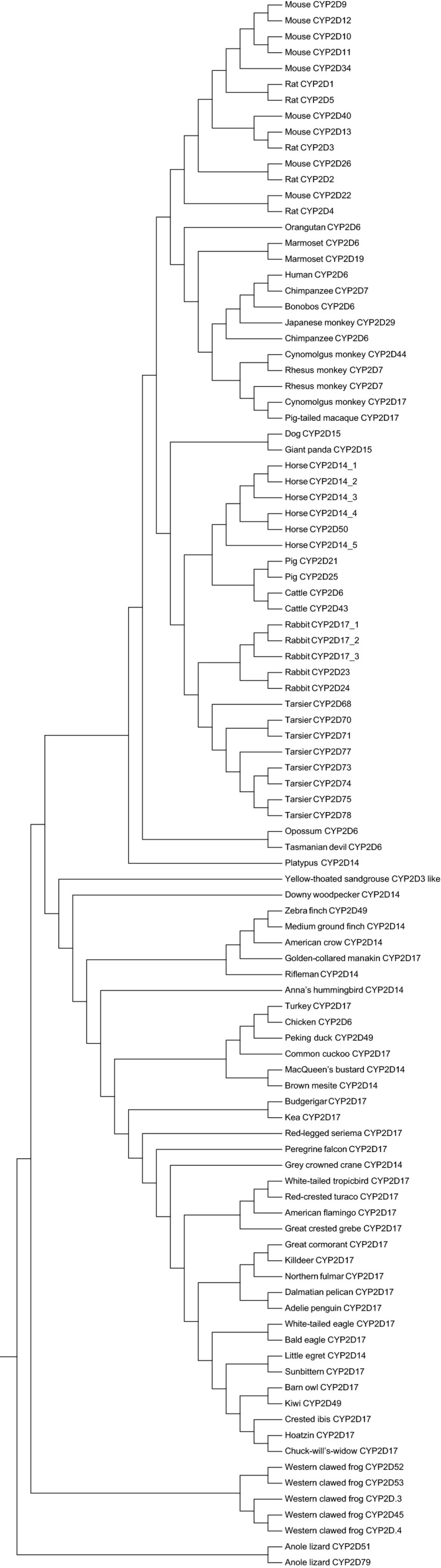
The NJ tree of all the studied enzymes to show the relationship between orthologs and paralogs of CYP2D subfamily. The enzymes of gorilla were excluded due to the failure to obtain CYP2D7. Amino acid sequence of mouse CYP2J6 was used as an outgroup

The NJ tree of CYP2D studied here is shown in Figure 3. The result of gene tree and species tree reconciliation revealed that 29 gene duplication events and 78 gene loss events happened in these species, and 18 species have no gain or loss (Table 1). When considering diet, the result demonstrated that gene duplication or loss events were not correlated with diet. For example, when the number of duplication events was 0 or equal to or greater than 4, the diet was carnivorous, herbivorous, or omnivorous. In addition, when the number of loss events was 0, 1 or 2, the species was carnivorous, herbivorous, or omnivorous.

**Table 1:** Times of gene duplication and loss events in CYP2D subfamily of the species studied. Diet information is also listed here for comparison

Species	Duplications	Losses	Diet
Brown_mesite	0	1	O
Orangutan	0	2	O
MacQueen's_bustard	0	1	O
Tasmanian_devil	0	0	O
Red‐crested turaco	0	1	H
Sunbittern	0	1	C
Mouse	4	0	O
Human	0	1	O
Cynomolgus monkey	0	1	O
Little egret	0	1	C
Giant panda	0	0	H
Peking duck	0	0	O
Kea	0	0	O
Budgerigar	0	0	H
Red‐legged seriema	0	3	C
Crested ibis	0	2	C
Anole lizard	1	1	C
Gray crowned crane	0	1	O
Platypus	0	0	C
Medium ground finch	0	0	H
Marmoset	1	5	O
Opossum	0	0	O
Pig‐tailed macaque	0	3	H
White‐tailed tropicbird	0	1	C
Rat	1	1	O
Turkey	0	0	O
Japanese monkey	0	2	H
Killdeer	0	1	O
Hoatzin	0	2	H
Downy woodpecker	0	1	O
Yellow‐throated sandgrouse	0	1	H
Dalmatian pelican	0	1	C
Tarsier	7	6	C
American crow	0	0	O
Chimpanzee	0	0	O
Horse	5	1	H
Rifleman	0	0	C
Barn owl	0	1	C
Great crested grebe	0	1	C
Golden‐collared manakin	0	0	O
Adelie penguin	0	1	C
White‐tailed eagle	0	0	C
Rabbit	4	1	H
Great cormorant	0	3	C
Rhesus monkey	0	2	O
Peregrine falcon	0	2	C
Chuck‐will's‐widow	0	1	C
Bonobos	0	4	H
Kiwi	0	14	C
Bald eagle	0	0	C
Cattle	1	1	H
Chicken	0	0	O
Common cuckoo	0	2	C
Pig	1	0	O
Western clawed frog	4	1	C
American flamingo	0	1	C
Dog	0	0	C
Zebra finch	0	0	H
Anna's hummingbird	0	1	H
Northern fulmar	0	1	C
Total	29	78	

## 4 DISCUSSION

This study comprehensively collected information on *CYP2D* gene numbers across vertebrates with different diets. In total, the information on *CYP2D* gene numbers was obtained from 73 species, including 22 mammals from 9 orders, 49 birds from 35 orders, 1 reptile, and 1 amphibian. The details are shown in Figure 1 and the Supporting Information. From statistical analyses of the relationship between *CYP2D* gene number and dietary preferences, the results suggested that although in some herbivorous species (for example, the horse and rabbit), *CYP2D* genes showed expansion, this trend cannot be extrapolated to all vertebrates. In other words, the number of *CYP2D* genes did not vary with feeding habits. A number of reasons could account for the results, several of which are likely, as follows. First, according to their substrates, P450 enzymes can be sorted into either the biosynthesis or detoxification type, the latter of which contains the CYP1‐4 families (Gotoh, 2012; Kawashima & Satta, 2014), and the *CYP2D* subfamily is just one member of the *CYP2* family responsible for toxin detoxification. Thus, the contribution of other *CYP2* genes or other *CYP* families may affect the relationship between *CYP2D* gene number and dietary preferences. A previous study that analyzed the relationship between the number of *CYP2* genes and diet in birds discovered that in migratory birds, omnivores had a higher number of *CYP2* genes than carnivores and herbivores (Almeida et al., 2016). In a preliminary analysis using the data from Thomas (2007), the same trend was observed in mammals (data not shown). Both of these studies indicated that the gene numbers of the whole *CYP2* family rather than those of only the *CYP2D* subfamily are associated with feeding habits. Second, Thomas (2007) suggested that throughout vertebrate evolution, *CYP* genes, which encode CYP450 enzymes acting upon exogenous chemicals, underwent active duplication and loss, mirroring numerous lineage‐ and species‐specific gene expansions. In addition, Sezutsu, Le Goff, and Feyereisen (2013) proposed that lineage‐specific expansions in *CYP* subfamilies are reflected in the distribution of *CYP* gene numbers within families and subfamilies. Thus, it is likely that *CYP2D* gene expansion did not happen at the level of vertebrates but at the species‐ and lineage‐specific levels. To test this hypothesis, a mammal group and a bird group were analyzed separately, but the result demonstrated that *CYP2D* gene copy numbers did not undergo lineage‐specific expansion along with diet. Thus, the pattern of evolution by birth‐ and death‐ of *CYP2D* genes appears to be more complex than previously appreciated. Third, other mechanisms can help to reduce the load of detoxification, which may result in smaller *CYP2D* gene numbers. For instance, for the number of *CYP2D* functional genes, an obvious difference is present between rodents and humans, with 9 and 1 genes, respectively. It is likely that for the mouse, the need for detoxification of potentially abundant toxins in the diet makes it necessary to keep several *CYP2D* genes active, whereas for human, the intellectual capability of avoiding the consumption of noxious substances and the passing on of information on appropriate food between generations can lead to the loss of selective pressure in maintaining the genes active (Ingelman‐Sundberg, 2005). Fourth, based on a comparison of several non‐human primates and human, the similarity range of CYP2D amino acid between non‐human primate and human is 90%–98% (He et al., 2016). In addition, different similarities will have different chances to substrate. Thus, no correlation between gene number and dietary preferences at the subfamily level could be attributed to the differentiation between amino acid similarities. Fifth, for gene duplication and loss events, Good et al. (2014) suggested that the rate of amino acid replacement was correlated with the number of P450 duplications and that gene loss could be due to the lower chemical diversity in narrower niches. In general, carnivores and herbivores are specialists, and omnivores are generalists; thus, the omnivores are confronted with more diverse chemicals, and accordingly, the number of duplications in omnivores should be greater than that in carnivores and herbivores do. However, in this study, the numbers of gene duplications and losses were not linked to dietary preferences. For example, when the number of gene duplication events was greater than or equal to 4, the feeding preferences were carnivorous (tarsier, western clawed frog), herbivorous (rabbit, horse), or omnivorous (mouse), and such a case also existed for gene loss events. Further, the conclusion of this study agreed with previous research (Sezutsu et al., 2013) proposing that the gain or loss of *CYP* genes did not rely on the ecological traits or life history traits of organisms, and thus, natural selection would not be predicted to be the determinant of *CYP* gene distribution. Finally, differences in how to deal with plant toxins might not be primarily ascribed to differentiation in the copy number of genes but instead to their isoforms, mutations, or regulation. In addition, the variation in CYP enzyme activity among different species may also affect the detoxification of diet (Rainio, Kanerva, Wahlberg, Nikinmaa, & Eeva, 2012). In sum, the gene expansion of the *CYP2D* subfamily is complex, and uncovering what truly acts as the driving force of *CYP2D* gene subfamily expansion still needs further investigation.

## 5 CONCLUSION

This study explored the association between the number of *CYP2D* subfamily genes and dietary preference and examined whether the number of genes varied according to diet. The results failed to conclude that *CYP2D* gene expansion was linked with diet, which means that feeding habit was not a driving force for *CYP2D* gene expansion. The reasons for *CYP2D* gene expansion are complicated, and the contribution of other *CYP* genes, other mechanisms reducing the load of detoxification, and differentiation between amino acid similarities may affect the relationship between *CYP2D* gene number and dietary preferences. The exact mechanism of the expansion still needs further study. As the genomes of increasing numbers of species are being sequenced, *CYP* gene data will increase, and further studies aiming to solve this problem are expected to be carried out.

## AUTHOR CONTRIBUTIONS

Ping Feng conceived and designed the experiments, performed the experiments, analyzed the data, contributed reagents/materials/analysis tools, prepared figures and/or tables, authored or reviewed drafts of the paper, approved the final draft. Zhijun Liu helped to collect data, provided suggestions for this study, and approved the final draft.

## DATA ACCESSIBILITY

The authors confirm that all data underlying the findings are fully available without restriction. All relevant data are presented in the paper and its Supporting Information.
