# Supplementary material for: Complex gene expansion of the CYP2D gene subfamily
Source: Ecol Evol. 2018 Oct 12;8(22):11022–30. doi: 10.1002/ece3.4568 (PMC6262912; doi:10.1002/ece3.4568)
Supplement: Supplementary file 1 [file ECE3-8-11022-s001.docx]

**Supplementary Material** The dietary preferences, *CYP2D* gene numbers, and other basic information of the species used in this study.

| Class | Order | Common Name | Species Name | Diet | Reference 1 | Number of total/intact/pseudo- gene | Reference 2 |
| --- | --- | --- | --- | --- | --- | --- | --- |
| Mammalia | Primates | Cynomolgus monkey | *Macaca fascicularis* | O | http://animaldiversity.org/ | 2/2/0 | [He et al. (2016](#_ENREF_7)); Chie Emoto et al (2013) |
|  |  | Japanese monkey | *Macaca fuscata* | H | http://animaldiversity.org/ | 1/1/0 | [He et al. (2016](#_ENREF_7)) |
|  |  | Pig-tailed macaque | *Macaca nemestrina* | H | http://animaldiversity.org/ | 1/1/0 | [He et al. (2016](#_ENREF_7)) |
|  |  | Rhesus monkey | *Macaca mulatta* | O | http://animaldiversity.org/ | 2/2/0 | Nelson (2009) |
|  |  |  |  |  |  | 2/2/0 | [He et al. (2016](#_ENREF_7)) |
|  |  | Bonobos | *Papio anubis* | H | http://animaldiversity.org/ | 2/1/1 | [He et al. (2016](#_ENREF_7)) |
|  |  | Human | *Homo sapiens* | O | http://animaldiversity.org/ | 3/1/2 | Nelson (2009) |
|  |  | Chimpanzee | *Pan troglodytes* | O | http://animaldiversity.org/ | 3/2/1 | [He et al. (2016](#_ENREF_7)) ; Nelson (2009) |
|  |  | Gorilla | *Gorilla gorilla* | H | http://animaldiversity.org/ | 3/2/1 | [He et al. (2016](#_ENREF_7)) |
|  |  | Orangutan | *Pongo pygmaeus* | O | Marriner and Drickamer (1994) | 3/1/2 | [He et al. (2016](#_ENREF_7)) |
|  |  | Marmoset | *Callithrix jacchus* | O | Bailey and Coe (2002) | 3/2/1 | [He et al. (2016](#_ENREF_7)) |
|  |  | Tarsier | *Tarsius syrichta* | C | Butynski (1982) | 11/8/3 | Nelson (2009) |
|  | Rodentia | Mouse | *Mus musculus* | O | Badan (1986) | 9/9/0 | Nelson et al. (2004); Nelson (2009) |
|  |  | Rat | *Rattus norvegicus* | O | Glendinning (1994) | 5/5/0 | Nelson (2009); Gonzalez and Nebert (1990); Yasukochi and Satta (2011) |
|  | Lagomorpha | Rabbit | *Oryctolagus cuniculus* | H | Glendinning (1994) | 5/5/0 | [Yasukochi and Satta (2011](#_ENREF_39)) |
|  | Carnivora | Dog | *Canis lupus* | C | Glendinning (1994) | 1/1/0 | Nelson (2009) |
|  |  | Giant panda | *Ailuropoda melanoleuca* | H | http://animaldiversity.org/ | 1/1/0 | [Yasukochi and Satta (2011](#_ENREF_39)) |
|  | Perissodactyla | Horse | *Equus caballus* | H | http://animaldiversity.org/ | 6/6/0 | [Yasukochi and Satta (2011](#_ENREF_39)) |
|  | Cetartiodactyla | Pig | *Sus scrofa* | O | Schley and Roper (2003) | 2/2/0 | Nelson (2009) |
|  |  | Cattle | *Bos Taurus* | H | http://animaldiversity.org/ | 2/2/0 | Nelson (2009) |
|  | Didelphimorphia | Opossum | *Monodelphis domestica* | O | Joly et al. (2012) | 1/1/0 | Nelson (2009) |
|  | Dasyuromorphia | Tasmanian devil | *Sarcophilus harrisii* | O | http://animaldiversity.org/ | 1/1/0 | Nelson (2009) |
|  | Monotremata | Platypus | *Ornithorhynchus anatinus* | C | Faragher et al. (1979) | 1/1/0 | Nelson (2009) |
| Aves | Passeriformes | Zebra finch | *Taeniopygia guttata* | H | http://animaldiversity.org/ | 1/1/0 | Almeida A et al. (2016) ; Nelson (2009) |
|  |  | Medium ground finch | *Geospiza fortis* | H | Scott (1996) | 1/1/0 | Almeida A et al. (2016) |
|  |  | American crow | *Corvus brachyrhynchos* | O | http://animaldiversity.org | 1/1/0 | Almeida A et al. (2016) |
|  |  | Golden-collared manakin | *Manacus vitellinus* | O | Worthington (1989) | 1/1/0 | Almeida A et al. (2016) |
|  |  | Rifleman | *Acanthisitta chloris* | C | Oliver (1955) | 1/1/0 | Almeida A et al. (2016) |
|  | Psittaciformes | Budgerigar | *Melopsittacus undulatus* | H | http://animaldiversity.org | 1/1/0 | Almeida A et al. (2016) |
|  |  | Kea | *Nestor notabilis* | O | http://animaldiversity.org | 1/1/0 | Almeida A et al. (2016) |
|  | Falconiformes | Peregrine falcon | *Falco peregrinus* | C | http://animaldiversity.org | 1/1/0 | Almeida A et al. (2016) |
|  | Cariamiformes | Red-legged seriema | *Cariama cristata* | C | del Hoyo et al. (1992) | 1/1/0 | Almeida A et al. (2016) |
|  | Coraciiformes | Carmine bee-eater | *Merops nubicus* | C | Hoi et al. (2015) | 0/0/0 | Almeida A et al(2016) |
|  | Piciformes | Downy woodpecker | *Picoides pubescens* | O | http://animaldiversity.org | 1/1/0 | Almeida A et al. (2016) |
|  | Bucerotiformes | Rhinoceros hornbill | *Buceros rhinoceros* | H | http://animaldiversity.org | 0/0/0 | Almeida A et al. (2016) |
|  | Trogoniformes | Bar-tailed trogon | *Apaloderma vittatum* | O | del Hoyo et al. (1992) | 0/0/0 | Almeida A et al. (2016) |
|  | Leptosomiformes | Cuckoo roller | *Leptosomus discolor* | C | del Hoyo et al. (1992) | 0/0/0 | Almeida A et al. (2016) |
|  | Coliiformes | Speckled mousebird | *Colius striatus* | H | Roberts et al. (2005) | 0/0/0 | Almeida A et al. (2016) |
|  | Strigiformes | Barn owl | *Tyto alba* | C | http://animaldiversity.org | 1/1/0 | Almeida A et al. (2016) |
|  | Accipitriformes | White-tailed eagle | *Haliaeetus albicilla* | C | del Hoyo et al. (1992) | 1/1/0 | Almeida A et al. (2016) |
|  |  | Bald eagle | *Haliaeetus leucocephalus* | C | http://animaldiversity.org | 1/1/0 | Almeida A et al. (2016) |
|  |  | Turkey vulture | *Cathartes aura* | C | http://animaldiversity.org | 0/0/0 | Almeida A et al. (2016) |
|  | Pelecaniformes | Dalmatian pelican | *Pelecanus crispus* | C | del Hoyo et al. (1992) | 1/1/0 | Almeida A et al. (2016) |
|  |  | Little egret | *Egretta garzetta* | C | Goutner et al. (1997) | 1/1/0 | Almeida A et al. (2016) |
|  |  | Crested ibis | *Nipponia nippon* | C | del Hoyo et al. (1992) | 1/1/0 | Almeida A et al. (2016) |
|  |  | Great cormorant | *Phalacrocorax carbo* | C | http://animaldiversity.org | 1/1/0 | Almeida A et al. (2016) |
|  | Sphenisciformes | Emperor penguin | *Aptenodytes forsteri* | C | http://animaldiversity.org | 0/0/0 | Almeida A et al(2016) |
|  |  | Adelie penguin | *Pygoscelis adeliae* | C | http://animaldiversity.org | 1/1/0 | Almeida A et al. (2016) |
|  | Procellariiformes | Northern fulmar | *Fulmarus glacialis* | C | http://animaldiversity.org | 1/1/0 | Almeida A et al. (2016) |
|  | Gaviiformes | Red-throated loon | *Gavia stellata* | C | http://animaldiversity.org | 0/0/0 | Almeida A et al. (2016) |
|  | Phaethontiformes | White-tailed tropicbird | *Phaethon lepturus* | C | Schreiber (1991) | 1/1/0 | Almeida A et al. (2016) |
|  | Eurypygiformes | Sunbittern | *Eurypyga helias* | C | Knowlton (1909) | 1/1/0 | Almeida A et al. (2016) |
|  | Charadriiformes | Killdeer | *Charadrius vociferus* | O | http://animaldiversity.org | 1/1/0 | Almeida A et al. (2016) |
|  | Gruiformes | Grey crowned crane | *Balearica regulorum* | O | http://animaldiversity.org | 1/1/0 | Almeida A et al. (2016) |
|  | Opisthocomiformes | Hoatzin | *Ophisthocomus hoazin* | H | http://animaldiversity.org | 1/1/0 | Almeida A et al. (2016) |
|  | Caprimuigiformes | Anna's hummingbird | *Calypte anna* | H | http://animaldiversity.org | 1/1/0 | Almeida A et al. (2016) |
|  |  | Chimney swift | *Chaetura pelagica* | C | http://animaldiversity.org | 0/0/0 | Almeida A et al. (2016) |
|  |  | Chuck-will's-widow | *Antrostomus carolinensis* | C | http://animaldiversity.org | 1/1/0 | Almeida A et al. (2016) |
|  | Otidiformes | Macqueen's bustard | *Chlamydotis macqueenii* | O | Perrins (2009) | 1/1/0 | Almeida A et al. (2016) |
|  | Musophagiformes | Red-crested turaco | *Tauraco erythrolophus* | H | del Hoyo et al. (1992) | 1/1/0 | Almeida A et al. (2016) |
|  | Cuculiformes | Common cuckoo | *Cuculus canorus* | C | http://animaldiversity.org | 1/1/0 | Almeida A et al. (2016) |
|  | Mesitornithiformes | Brown mesite | *Mesitornis unicolor* | O | del Hoyo et al. (1992) | 1/1/0 | Almeida A et al. (2016) |
|  | Pteroclifornes | Yellow-thoated sandgrouse | *Pterocles gutturalis* | H | Campbell and Lack. (1985) | 1/1/0 | Almeida A et al. (2016) |
|  | Columbiformes | Domestic pigeon | *Columba livia* | H | http://animaldiversity.org | 1/0/1 | Almeida A et al. (2016) |
|  | Phoenicopteriformes | American flamingo | *Phoenicopterus ruber* | C | http://animaldiversity.org | 1/1/0 | Almeida A et al. (2016); Zhang G et al. (2014) |
|  | Podicipediformes | Great crested grebe | *Podiceps cristatus* | C | http://animaldiversity.org | 1/1/0 | Almeida A et al. (2016) |
|  | Galliformes | Turkey | *Meleagris gallopavo* | O | http://animaldiversity.org | 1/1/0 | Almeida A et al. (2016) ; Nelson (2009) |
|  |  | Chicken | *Gallus gallus* | O | http://animaldiversity.org | 1/1/0 | Almeida A et al. (2016); Nelson (2009) |
|  | Anseriformes | Peking duck | *Anas platyrhynchos* | O | http://animaldiversity.org | 1/1/0 | Almeida A et al. (2016) ; Nelson (2009) |
|  | Tinamiformes | White-throated tinamou | *Tinamus guttatus* | H | Perrins (2009) | 0/0/0 | Almeida A et al. (2016) |
|  | Apterygiformes | Kiwi | *Apteryx australis* | C | http://animaldiversity.org | 1/1/0 | Nelson (2009) |
|  | Struthioniformes | Common ostrich | *Struthio camelus* | H | http://animaldiversity.org | 0/0/0 | Almeida A et al. (2016) |
| Reptilia | Squamata | Anole lizard | *Anolis carolinensis* | C | http://animaldiversity.org/ | 3/2/1 | Nelson (2009) |
| Amphibians | Anura | Western clawed frog | *Xenopus tropicalis* | C | Roedel et al. (2010) | 5/5/0 | Nelson (2009) |

Note: References1 is for the dietary preference, and references2 is for gene number.

**References 1**

1 Marriner, L. & Drickamer, L. 1994. Factors influencing stereotyped behavior of primates in a zoo. *Zoo. Biol.* **13**: 267-275.

2 Bailey, M.T. & Coe, C.L. 2002. Intestinal microbial patterns of the common marmoset and rhesus macaque. *Comp. Biochem. Physiol. A Comp. Physiol.* **133**: 379-388.

3 Butynski, T.M. 1982. Vertebrate predation by primates: a review of hunting patterns and prey. *J. Hum. Evol.* **11**: 421-430.

4 Badan, D. 1986. Diet of the house mouse (Mus musculus L.) in two pine and a Kauri forest. *New Zeal. J. Ecol.* **9**: 137-142.

5 Glendinning, J.I. 1994. Is the bitter rejection response always adaptive? *Physiol. Behav.* **56**: 1217-1227.

6 Schley, L. & Roper, T.J. 2003. Diet of wild boar Sus scrofa in Western Europe, with particular reference to consumption of agricultural crops. *Mammal. Rev.* **33**: 43-56.

7 Joly, M., Scheumann, M. & Zimmermann, E. 2012. Posture does not matter! paw usage and grasping paw preference in a small-bodied rooting quadrupedal mammal. *PLoS One* **7**: e38228.

8 Faragher, R., Grant, T. & Carrick, F. 1979. Food of the platypus (Ornithorhynchus anatinus) with notes on the food of brown trout (Salmo trutta) in the Shoalhaven River, NSW. *Aust. J. Ecol.* **4**: 171-179.

9 Scott, T.A. 1996. *Concise encyclopedia biology*. Berlin, Walter de Gruyter.

10 Oliver, W.R.B. 1955. *New Zealand birds*. 2nd ed. Wellington, AH & AW Reed.

11 del Hoyo, J., Elliott, A., Sargatal, J. & Cabot, J. 1992 *Handbook of the birds of the world*. Barcelona, Lynx Edicions.

12 Hoi, H., Kristofik, J. & Darolova, A. 2015. All you can eat: is food supply unlimited in a colonially breeding bird? *Ecol. Evol.* **5**:

450-458.

13 Roberts, A., Hockey, P.A.R., Dean, W.R.J. & Ryan, P. 2005. *Roberts’ birds of Southern Africa*. 7th ed. Cape Town, Trustees of the

John Voelcker Bird Book Fund.

14 Goutner, V. & Furness, R.W. 1997. Mercury in feathers of little egret Egretta garzetta and night heron Nycticorax nycticorax

chicks and in their prey in the Axios Delta, Greece. *Arch. Environ. Contam. Toxicol.* **32**: 211-216.

15 Schreiber, E.A. 1991. *Encyclopaedia of Animals: Birds*. London, Merehurst Press.

16 Knowlton, F.H. & Lucas, F.A. 1909. *Birds of the World: A popular account*. Holt.

17 Perrins, C.M. 2009. *The Princeton encyclopedia of birds*. Princeton, N.J., Princeton University Press.

18 Campbell, B., Lack, E. & British Ornithologists' Union. 1985. *A dictionary of birds*. Vermillion, S.D., Published for the British

Ornithologists' Union by Buteo Books.

19 Roedel, M.O., Vredenburg, V., Mahoney, M.J., Tunstall, T. &Whittaker, K. 2010. Xenopus tropicalis. AmphibiaWeb. Retrieved 2013-12-06.

**References 2**

1 He, Z. X., Chen, X. W., Yang, Y. & Zhou, S. F. 2016. A comparison of non-human primate cytochrome P450 2D members and the implication in drug discovery. Curr. Drug Metab. **17**: 520-527.

2 Emoto, C., Yoda, N., Uno, Y., et al. 2013. Comparison of P450 enzymes between cynomolgus monkeys and humans:P450

identities, protein contents, kinetic parameters, and potential for inhibitory profiles. Curr. Drug Metab. **14**: 239-252.

3 Yasukochi, Y. & Satta, Y. 2011. Evolution of the CYP2D gene cluster in humans and four non-human primates. Genes Genet. Syst. **86** :109.

4 Yasukochi, Y. & Satta, Y. 2015. Molecular evolution of the cyp2d subfamily in primates: purifying selection on substrate recognition sites without the frequent or long-tract gene conversion. *Genome Biol. Evol.* **7**: 1053-1067.

5 Nelson, D.R. 2009. The cytochrome p450 homepage. *Hum. Genomics* **4**: 59-65.

6 Nelson, D.R., Zeldin, D.C., Hoffman, S.M., Maltais, L.J., Wain, H.M. & Nebert, D.W. 2004. Comparison of cytochrome P450 (CYP) genes from the mouse and human genomes, including nomenclature recommendations for genes, pseudogenes and alternative-splice variants. Pharmacogenetics **14**: 1-18.

7 Gonzalez, F.J. & Nebert, D.W. 1990. Evolution of the P450 gene superfamily: animal-plant 'warfare', molecular drive and human genetic differences in drug oxidation. Trends Genet. **6**: 182-186.

8 Almeida, D., Maldonado, E., Khan, I., Silva, L., Gilbert, M.T., Zhang, G. *et al.* 2016. Whole-genome identification, phylogeny, and evolution of the cytochrome P450 family 2 (CYP2) subfamilies in birds. *Genome Biol. Evol.* **8**: 1115-1131.
